# Integrating transcriptomic and metabolomic analysis in roots of wild soybean seedlings in response to low-phosphorus stress

**DOI:** 10.3389/fpls.2022.1006806

**Published:** 2022-11-17

**Authors:** Mingxia Li, Ji Zhou, Xianyu Lang, Defu Han, Yongjun Hu, Yinan Ding, Guangye Wang, Jixun Guo, Lianxuan Shi

**Affiliations:** ^1^ School of Life Sciences, Changchun Normal University, Changchun, China; ^2^ Land Consolidation and Rehabilitation Centre, The Ministry of Land and Resources, Beijing, China; ^3^ Institute of Grassland Science, Northeast Normal University, Key Laboratory of Vegetation Ecology, Ministry of Education, Changchun, China

**Keywords:** adaptations, metabolomics, phosphorus deficiency, transcriptomics, wild soybean

## Abstract

**Introduction:**

Plants undergo divergent adaptations to form different ecotypes when exposed to different habitats. Ecotypes with ecological adaptation advantages are excellent germplasm resources for crop improvement

**Methods:**

his study comprehensively compared the differences in morphology and physiological mechanisms in the roots of two different ecotypes of wild soybean (Glycine soja) seedlings under artificially simulated low-phosphorus (LP) stress.

**Result:**

The seedlings of barren-tolerant wild soybean (GS2) suffered less damage than common wild soybean (GS1). GS2 absorbed more phosphorus (P) by increasing root length. In-depth integrated analyses of transcriptomics and metabolomics revealed the formation process of the ecological adaptability of the two different ecotypes wild soybean from the perspective of gene expression and metabolic changes. This study revealed the adaptation process of GS2 from the perspective of the adaptation of structural and molecular metabolism, mainly including: (1) Enhancing the metabolism of phenolic compounds, lignin, and organic acid metabolism could activate unavailable soil P; (2) Up-regulating genes encoding pectinesterase and phospholipase C (PLC) specifically could promote the reuse of structural P; (3) Some factors could reduce the oxidative damage to the membranes caused by LP stress, such as accumulating the metabolites putrescine and ascorbate significantly, up-regulating the genes encoding SQD2 (the key enzyme of sulfolipid substitution of phospholipids) substantially and enhancing the synthesis of secondary antioxidant metabolite anthocyanins and the AsA-GSH cycle; (4) enhancing the uptake of soil P by upregulating inorganic phosphate transporter, acid phosphatase ACP1, and purple acid phosphatase genes; (5) HSFA6b and MYB61 are the key TFs to resist LP stress.

**Discussion:**

In general, GS2 could resist LP stress by activating unavailable soil P, reusing plant structural P, rebuilding membrane lipids, and enhancing the antioxidant membrane protection system. Our study provides a new perspective for the study of divergent adaptation of plants.

## 1 Introduction

Phosphorus (P) is one of the important mineral nutrients for plant growth and development (Gamuyao et al., 2012). Due to the adsorption of metal cations and the activities of microorganisms, the effective P concentration that can be absorbed by plants from the soil is only 0.1–10 μmol·L^−1^. The solubility of phosphate in soil is very low and its diffusion rate is very slow, making it difficult for plants to use (Lambers et al, 2006; Shen et al., 2018), which often does not ensure plant productivity, resulting in plants in a state of low-phosphorus (LP) stress (Huang et al., 2008). In this case, supplementing basic knowledge of the effects of poor soil on plant genetics and physiological metabolism helps in understanding how plants have evolved by adapting to their habitat (Dool et al., 2022).

Soybean (*Glycine max*) has the highest protein content among existing crops, and is also the oil crop ranking first in oil production in the world. In addition to being an important component of soybean plants, P is also involved in enzymatic, metabolism, nodule nitrogen fixation, and other physiological and biochemical processes. In soybean production, P deficiency is more harmful than other stresses, so it is very important to improve soybean’s tolerance to LP stress and P absorption efficiency (Zhang et al., 2014). After a long period of natural selection, wild soybean (*Glycine soja*) has evolved many excellent traits and has a strong ability to tolerate barrenness, such as low nitrogen and salt stress (Li et al., 2018). As a relative of soybean, wild soybean is an important natural wild plant resource to breed soybeans tolerant to LP stress. Thus, clarifying the processes of gene expression and physiological and metabolic adaptations that occurred during the differentiation into different ecotypes of wild soybean for habitat adaptation can inform breeding programs and germplasm conservation strategies.

Under extreme P deficiency, plants adjust morphological structure, physiological metabolism, and break down structural P (e.g., membrane lipids and cell walls) to release organic P for reuse to maintain plant survival (Yang et al., 2016; Wei et al., 2020). The P in plants is mainly obtained from the soil P pool through the roots. In response to LP, root length increases as do the density of lateral roots and root hairs (Li et al., 2015). Increasing the accumulation of organic acids in roots and the secretion of organic acid anions and releasing soluble phosphates in the soil is an effective way for plants to adapt to P deficiency, and has been reported in white lupin (*Lupinus albus*), oats, alfalfa pigeon peas, common bean, rice, maize, barley, canola and radish (Lin et al., 2011; Chen and Liao, 2016; Zou et al., 2018). The accumulation of secondary antioxidants is of great significance for scavenging reactive oxygen species (ROS) and protecting membrane structure under P deficiency, which has been confirmed in wheat, sorghum, and switchgrass (Ding et al., 2021; Kumar et al., 2021). When P is insufficient, to ensure the normal function of cells, the enzyme SQD2 can make up for the lack of phospholipids and maintain the integrity of membrane structure (Yang et al., 2003; Mo et al., 2019). Based on previous studies, clarifying the mechanism of resistance of the wild soybean root system to P deficiency in soil is of great significance to improve the LP tolerance of cultivated soybean. This provides new ideas and methods for research on the adjustment of physiological metabolism and adaptive evolution of plants in adapting to different environments.

Our goal here is to clarify the reasons for evolutionary adaptability differences in different wild soybean ecotypes under natural conditions, to evaluate the hypothesis that the resistance of barren-tolerant wild soybean to LP stress depends on increasing root length, decomposing structural phosphorus, improving organic acid metabolism, and enhancing antioxidant system. This study compared differences in morphological structure, physiology metabolism, and gene expression in two ecotypes of wild soybean seedlings under artificially simulated LP stress by in-depth integrated analyses of transcriptomics and metabolomics. The objectives were to (1) ​identify differentially expressed genes (DEGs) in barren-tolerant wild soybean under LP stress, particularly associated with activating unavailable soil P, reusing plant structural P, rebuilding membrane lipids, and enhancing the antioxidant membrane protection system. (2) determine metabolites changes and key metabolic pathways caused by gene expression changes under LP stress. (3) reveal the key metabolic pathways, key genes and key metabolites of adaptation of barren-tolerant wild soybean to LP stress. This study could provide an important reference value for research on the adaptive evolution of plants and provides quantitative system parameters for resource evaluation.

## 2 Materials and methods

### 2.1 Plant materials, growth conditions, and plant harvest

Barren-tolerant wild soybean was obtained from 20 wild soybean varieties sampled on-site from saline-alkaline barren grassland in Tongyu County, Baicheng City, western Jilin Province. Tongyu is a county with an average annual precipitation of 350 mm, the soil feature of which is barren and saline alkali sandy soil. The best barren-tolerant wild soybeans were screened and identified from these varieties through a P-deficiency experiment. Meanwhile, common types of wild soybean were selected from those grown in nutrient-rich soils in Huinan County, Tonghua City, Southeast Jilin Province. Huinan is a county with an average annual precipitation of 737.4 mm, the soil feature of which is black humus and fertile. The ecological environment of Tongyu is more barren than Huinan. The resulting experimental materials were common wild soybean (“Huinan 06116”, GS1) and barren-tolerant wild soybean (“Tongyu 06311”, GS2). Seeds were uniformly sown in a mud film in a 14-cm diameter flowerpot and covered with clean sand, and then watered until germination. After germination, seedlings were irrigated with 1× Hoagland nutrient solution (with KH_2_PO_4_ concentration of 2000 μM). When the seedlings had their third compound leaves, the two ecotypes of wild soybean were randomly divided into control (CK) and LP stress groups, with 12 pots in each group. The CK group continued to be irrigated with 1× Hoagland nutrient solution, and the LP stress group was irrigated with 1× Hoagland nutrient solution containing 2.5 μM KH_2_PO_4_; the insufficient potassium in the LP stress group was supplemented with the same concentration of KCl. After 10 days of treatment, four pots were randomly selected from each group to harvest the seedling roots for transcriptomic analysis. After 14 days of treatment, four pots were randomly selected from each group to harvest the seedlings to determine growth parameters and ion contents. After 14 days of treatment, the remaining four pots from each group were used to harvest the seedling roots for metabonomic analysis. The experimental materials were cultivated in the outdoor test field of Northeast Normal University (Changchun City, Jilin Province, China). The average temperature during day and night was 26 ± 2°C and 18.5 ± 1.5°C, respectively; the relative humidity was 60.5%.

### 2.2 Measurement of growth parameters

The plant height, root length, and underground fresh and dry weights of seedlings of both wild soybean ecotypes were measured (Li et al., 2018). All measurements were repeated four times with four biological replicates per sample. The data obtained were analyzed using SAS 9.2 (NCSU, NC, USA).

### 2.3 Measurement of P contents

After collecting fresh samples of plant roots and drying them, 50 mg of each dry sample was placed in deionized water, boiled and soaked overnight, and centrifuged at 3000 × g for 15 min. The supernatant was collected then add deionized water to boil and centrifuge twice without overnight, and the supernatant was collected. The volume of the three supernatants was adjusted to 10 mL, and used to determine the P contents using inductively coupled plasma mass spectrometry (ICP-MS, iCAP RQ, Germany) (Zhu et al., 2021). Data of P content are presented as the mean ± standard deviation of three biological replicates. The data obtained were analyzed using SAS 9.2 (NCSU, NC, USA).

### 2.4 Transcriptomics analysis

#### 2.4.1 RNA extraction, library preparation, and Illumina sequencing

The total RNA of samples was extracted by Trizol method. The RNA concentration was measured using a NanoDrop 2000 (Thermo Scientific Inc., DE, USA). The RNA integrity was assessed using an RNA Nano 6000 Assay Kit on an Agilent Bioanalyzer 2100 system (Agilent Technologies, CA, USA). Sequencing libraries were generated using NEBNext®Ultra™ RNA Library Prep Kit for Illumina® (NEB, USA), and index codes were added to assign sequences to each sample. Clustering of the index-coded samples was performed on a cBot Cluster Generation System using TruSeq PE Cluster Kit v3-cBot-HS (Illumina, NEB, USA) according to the manufacturer’s instructions. After cluster generation, the library preparations were sequenced on an Illumina HiSeq platform. After the original number, a series of processing and analyses was performed to obtain clean reads, which were then mapped to the reference Williams 82 genome sequence based on good comparison efficiency.

#### 2.4.2 Data processing, screening, and functional analysis of differential genes

The DESeq R package (1.10.1) was used to analyze the expression in genes of the CK and LP stress groups of both experimental materials. Each experimental material was screened for differentially expressed genes (DEGs) using FDR < 0.05 and |log_2_FC| ≥ 1.

FPKM = cDNA fragments/mapped fragments (millions) × transcript length (kb) (Florea et al., 2013).

The FC in log_2_FC is fold change, which represents the ratio of expression between the samples of the LP and CK groups. Gene function was annotated based on the GO (Gene Ontology) database. The GO enrichment analysis of the DEGs was implemented using the GOseq R package based on a Wallenius non-central hyper-geometric distribution (Young et al., 2010). The DEGs related to metabolism were identified based on the Kyoto Encyclopedia of Genes and Genomes (KEGG) (http://www.genome.jp/keg).

#### 2.4.3 Quantitative real-time PCR validation

To ensure reliability of transcriptome analysis data, 10 DEGs randomly selected from both ecotypes of wild soybean separately were detected by real-time fluorescence quantitative PCR (qRT-PCR) to verify the data. Primer Premier 6 software (Premier Biosoft International, Palo Alto, CA, USA) was used to design primers. TBtools software (http://www.tbtools.com) was used to assess the specificity of primers, and the primer sequences of these genes are listed in **Supplementary material Table S1** (Schmittgen and Livak, 2008).

### 2.5 Metabolome analyses

#### 2.5.1 Root metabolite extraction and GC/MS untargeted metabolic profiling

Of frozen plant root samples, 100 ± 5 mg was transferred into a 2-mL centrifuge tube, extracted with 0.3 mL methanol and 0.1 mL chloroform, and 60 μL was added to ribitol-containing water as an internal standard. The pellet was ground in a 70-Hz grinding system (Jinxin Biotechnology Co., Ltd., Shanghai, China) for 5 min, then incubated at 70°C for 10 min, and centrifuged at 12,000 × g or 10 min at 4°C. Of the supernatant, 0.35 mL was placed in a 2-mL glass tube, dried in a vacuum at 30°C for 2 h, dissolved in 80 μL of methoxyamine hydrochloride (20 mg/mL pyridine solution), then placed in a vacuum concentrator and dried, and then placed in an oven (MKX-J1-10, Qingdao Microwave Technology Co., Ltd., Qingdao, China) at 37°C for 2 h. The sample was further derivatized with N,O-bis (trimethylsilyl)-trifluoroacetamide containing 1% trimethylchlorosilane (100 μL) at 70°C for 1 h. Then the derived sample was cooled to room temperature. The samples were used for gas chromatograph-mass spectrometer (GC/MS) analysis using a one-dimensional Agilent 7890 GC system coupled with a Pegasus HT time-of-flight MS. There were four biological replicates for each experimental material and each treatment.

#### 2.5.2 Metabolomics data analysis

The raw data were preprocessed with ChromaTOF software (version 2.12, 2.22, 3.34; LECO, St. Joseph, MI, USA). The LECO-Fiehn Rtx5 database was used for metabolite identification by matching the mass spectrum and retention index. Then, metabolite data were normalized according to Li et al. (2017) and imported into the SIMCA-P 13.0 software package (Umetrics, Umea, Sweden) for further data analysis, including principal component analysis (PCA), orthogonal partial least-squares discrimination analysis (OPLS-DA) and partial least squares discriminant analysis (PLS-DA). Finally, based on changes in metabolite concentration compared to the corresponding control, Student’s t-test (p < 0.05), VIP > 1, and similarity value > 700 were used to screen differential metabolites. Then, KEGG was used to analyze metabolites and construct metabolic pathways, and the MetaboAnalyst website (http://http://www.metaboanalyst.ca) was used to analyze the pathways (Xia et al., 2012).

#### 2.5.3 Integrated transcriptomics and metabolomics analysis

Pearson’s correlation coefficients among the DEGs and differential metabolites were calculated using MetaboAnalyst 3.0 (www.metaboanalyst.ca). The integration network was analyzed using Cytoscape (version 3.8.2).

## 3 Results

### 3.1 Changes in plant morphology and P content

**Figure 1:**
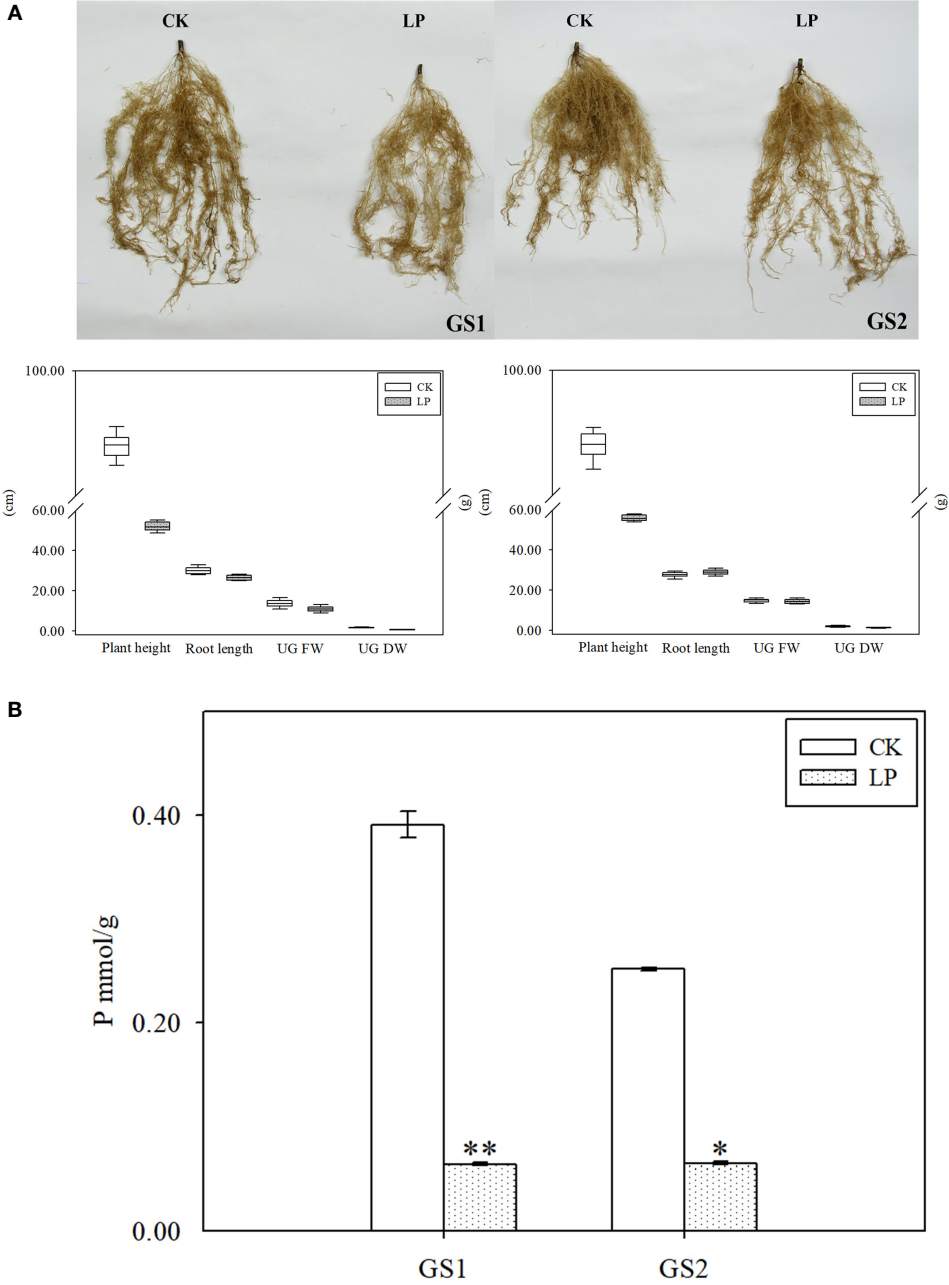
**(A)** Root phenotypes and **(B)** phosphorus content of roots of two ecotypes of wild soybean seedlings under control and low-phosphorus stress. GS1, common wild soybean; GS2, barren-tolerant wild soybean; CK, control group; LP, low-phosphorus stress group. * Significant at P < 0.05, ** significant at P < 0.01.

The plant height, root length, underground fresh weight and underground dry weight of the two ecotypes of wild soybean seedlings changed significantly under LP stress. Compared with CK, the roots of both ecotypes became sparse and lateral roots were significantly reduced (**Figure 1A**). Under LP stress, plant height, underground fresh weight, and underground dry weight in GS1 decreased by 0.81-fold (*p* < 0.05) and 0.32- and 1.31-fold (*p* < 0.01), respectively, and correspondingly in GS2 decreased by 0.70-fold (*p* < 0.05) and 0.05- and 0.66-fold (*p* < 0.05). The change of plant root length was opposite in the two ecotypes: root length decreased by 0.14-fold in GS1, but increased significantly by 0.06-fold in GS2 (**Table 1**). The changes of morphology showed significantly greater growth inhibition for GS1 than GS2 under LP stress. In addition, the P content in the seedling roots of common and barren-tolerant wild soybean under LP stress decreased significantly by 2.61-fold (*p* < 0.01) and 1.95-fold (*p* < 0.05), respectively (**Figure 1B**). The decline degree of P in GS1 was significantly higher than that in GS2.

**Table 1:** Effects of LP stress on growth parameters of two ecotypes of wild soybean seedlings.

	GS1		GS2		FD	
	CK	LP	CK	LP	GS1	GS2
Plant height (cm)	91	52	91	56	-0.81*	-0.70*
Root length (cm)	30	27.2	27.7	28.93	-0.14	0.06*
Underground fresh weight (g)	13.7	11	14.8	14.3	-0.32	-0.05
Underground dry weight (g)	1.73	0.7	2	1.27	-1.31**	-0.66

GS1, common wild soybean; GS2, barren-tolerant wild soybean; CK, control group; LP, low-phosphorus stress group. * Significant at P < 0.05, ** significant at P < 0.01. FD, fold changes that the change of growth parameters is represented by log_2_^(LP/CK)^.

### 3.2 Changes in transcription level

Transcriptomics sequencing produced a total of 88.67 Gb of clean data, and the clean data of each sample reached 6.68 Gb with the quality score 30 (Q30) base percentage being 93.96% and above. The comparison efficiency of the clean reads of each sample and the reference genome was within 89.01–93.48% (**Supplementary material Table S2**). Ten DEGs were randomly selected from the two ecotypes of wild soybean to perform qRT-PCR separately. The results were very similar to the transcriptome sequencing data, and so verified their accuracy (**Supplementary material Figure S1**).

**Figure 2:**
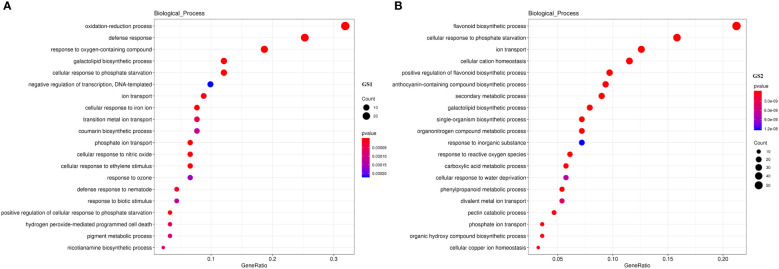
GO enriched biological process annotation of DEGs in **(A)** common and **(B)** barren-tolerant wild soybean. GS1, common wild soybean; GS2, barren-tolerant wild soybean.

Compared with CK, there were 340 DEGs in GS1 seedling roots (139 significantly up-regulated and 201 significantly down-regulated), 2948 DEGs in GS2 seedling roots (1688 significantly up-regulated and 1260 significantly down-regulated) under LP stress, and there were 108 common DEGs in roots of both ecotypes (**Supplementary material Figure S2**). There were many more DEGs in GS2 than GS1 seedling roots, showing that GS2 could more actively respond to and resist LP stress. The GO annotation analysis of DEGs showed that 145 and 1624 DEGs in biological processes were annotated to 86 and 399 items, respectively; 16 and 1285 DEGs in cell components were annotated to 16 and 40 items, respectively; and 75 and 1036 DEGs in molecular function were annotated to 51 and 206 entries in GS1 and GS2 seedling roots, respectively (items *p* value < 0.05) (**Supplementary Material Figure S3**). Further analysis of the DEGs in biological processes in GS1 roots showed that these were significantly mainly distributed in cellular response to phosphate starvation (GO:0016036, 11 DEGs), ion transport (GO:0006811, eight DEGs), coumarin biosynthetic process (GO:0009805, seven DEGs), phosphate ion transport (GO:0006817, six DEGs), and positive regulation of cellular response to phosphate starvation (GO:0080040, three DEGs) (**Figure 2A**; **Supplementary Material Table S3**). Further analysis of DEGs in biological processes in GS2 roots showed that the significant distribution was divided into two major parts: mineral nutrition and metabolic activities. Among them, mineral nutrients included cellular response to phosphate starvation (GO:0016036, 44 DEGs), ion transport (GO:0006811, 35 DEGs), cellular cation homeostasis (GO:0030003, 32 DEGs), phosphate ion transport (GO:0006817, 10 DEGs), cellular copper ion homeostasis (GO:0006878, nine DEGs), and metabolic activities, including flavonoid biosynthetic process (GO:0009813, 59 DEGs), positive regulation of flavonoid biosynthetic process (GO:0009963, 27 DEGs), anthocyanin-containing compound biosynthetic process (GO:0009718, 26 DEGs), secondary metabolic process (GO:0019748, 25 DEGs), response to ROS (GO:0000302, 17 DEGs), carboxylic acid metabolic process (GO:0019752, 16 DEGs), phenylpropanoid metabolic process (GO:0009698, 15 DEGs), and pectin catabolic process (GO:0045490, 13 DEGs) (**Figure 2B**, **Supplementary Material Table S3**). Thus, GS2 was more active than GS1 in response to LP stress at the level of mineral nutrition and metabolism. The DEGs related to the acquisition of P and transportation of phosphate, including PHT1, ACP and PAP were significantly up-regulated in GS2 (**Table 2**). Among them, the DEGs Glyma.03G162800.Wm82.a2.v1, which is responsible for encoding inorganic phosphate transporter 1-2 (PHT1-2) and Glyma.10G006700.Wm82.a2.v1, which is responsible for encoding inorganic phosphate transporter 1-3 (PHT1-3), were significantly up-regulated by 1.53- and 2.31-fold in GS2. The acid phosphatase ACP1 (ACP1) gene Glyma.08G195100.Wm82.a2.v1 was significantly up-regulated by 2.82-fold in GS2 seedling roots. Furthermore, the purple acid phosphatase genes, Glyma.12G007500.Wm82.a2.v1 (PAP36), Glyma.09G229200.Wm82.a2.v1 (PAP14), and Glyma.10G071000.Wm82.a2.v1 (LOC100782755) were significantly upregulated by 1.87-, 1.67- and 1.80-fold in GS2 seedling roots (**Supplementary material Table S4**).

**Table 2:** The expression of typical LP stress induced genes of DEGs in roots of two ecotypes wild soybean seedlings under LP.

	Name	ID	FPKM		FC	Description
			CK	LP		
**GS1**	PHT1-3	Glyma.10G006700.Wm82.a2.v1	22.65	55.64	1.19	Inorganic phosphate transporter 1-3
	PAP36	Glyma.12G007500.Wm82.a2.v1	2.55	4.29	0.64	purple acid phosphatase 36
	LOC100782755	Glyma.10G071000.Wm82.a2.v1	2.98	11.61	1.23	purple acid phosphatase 22
**GS2**	PHT1-2	Glyma.03G162800.Wm82.a2.v1	12.25	34.56	1.53	inorganic phosphate transporter 1-2
	PHT1-3	Glyma.10G006700.Wm82.a2.v1	18.19	91.31	2.31	Inorganic phosphate transporter 1-3
	ACP1	Glyma.08G195100.Wm82.a2.v1	0.58	4.1	2.82	acid phosphatase ACP1
	PAP36	Glyma.12G007500.Wm82.a2.v1	1.14	4.53	1.87	purple acid phosphatase 36
	PAP14	Glyma.09G229200.Wm82.a2.v1	0.6	2.27	1.67	purple acid phosphatase
	LOC100782755	Glyma.10G071000.Wm82.a2.v1	1.18	5.58	1.8	purple acid phosphatase 22

The KEGG enrichment analysis for 2948 DEGs showed that 591 DEGs were enriched in 111 pathways, among which the top 20 metabolic pathways with p < 0.05 are shown in **Supplementary material Figure S4**. Mapping the annotated DEGs to the KEGG database showed that four, two, one, one, and four transcripts were significantly enriched in phenylpropanoid biosynthesis (Ko00940), pentose phosphate pathway (Ko00030), glutathione metabolism (Ko00480), glycerolipid metabolism (Ko00561), and pentose and glucuronate interconversions (ko00040), respectively, in GS1 roots (**Table 3**, **Supplementary material Figure S4**.). Mapping the annotated DEGs to the KEGG database showed that 31, 13, 4, 6, 3, 4, 2, 1, and 10 transcripts were significantly enriched in phenylpropanoid biosynthesis (ko00940); flavonoid biosynthesis (ko00941); phenylalanine, tyrosine, and tryptophan biosynthesis (ko00400); pentose phosphate pathway (ko00030); glycine, serine, and threonine metabolism (ko00260); glutathione metabolism (ko00480); glycerolipid metabolism (ko00561); pentose and glucuronate interconversions (ko00040); and glycerophospholipid metabolism (ko00564), respectively, in GS2 roots (**Table 4**; **Supplementary Material Figure S4**.). Related to lignin synthesis enzyme cinnamyl-alcohol dehydrogenase (CAD), the DEGs Glyma.03G208200.Wm82.a2.v1 and Glyma.09G277900.Wm82.a2.v1 were up-regulated 1.28- and 1.02-fold in GS1 roots, respectively, and Glyma.08G145900.Wm82.a2.v1 and Glyma.14G221200.Wm82.a2.v1 were up-regulated 1.30- and 1.11-fold in GS2 roots, respectively. There were three and 28 DEGs related to the lignin synthesis enzyme peroxidase significantly up-regulated in GS1 and GS2 roots, respectively, with significantly greater up-regulation in GS2 than GS1 roots. Related to glutathione-glutathione disulfide (GSH-GSSH) metabolism, the glutathione peroxidase (gpx) gene Glyma.11G024000.Wm82.a2.v1 was significantly down-regulated 1.10-fold in GS1 roots, but Glyma.02G149100.Wm82.a2.v1 in GS2 roots was up-regulated 1.10-fold. In addition, related to ascorbate (AsA)–GSH cycle ornithine decarboxylase, DEGs Glyma.04G020200.Wm82.a2.v1 and Glyma.06G020300.Wm82.a2.v1 were up-regulated 1.71- and 1.71-fold, respectively, and L-ascorbate peroxidase gene Glyma.14G177200.Wm82.a2.v1 was up-regulated 1.21-fold in GS2 roots, but there was no change in GS1. Related to the enzyme SQD2 that promotes the substitution of sulfolipids for phospholipids, gene Glyma.03G078300.Wm82.a2.v1 was up-regulated 1.10-fold in GS1 roots and Glyma.03G078300.Wm82.a2.v1 was up-regulated 1.71-fold in GS2 roots, showing that GS2 had a stronger sulfolipid–phospholipid substitution effect. There were three and 10 DEGs, related to pectin esterase that catalyzes the reaction of cell-wall pectin, significantly up-regulated in GS1 and GS2 roots, respectively, with significantly greater up-regulation in GS2 than in GS1. In addition, DEGs related to anthocyanin synthesis, flavanone 4-reductase (DFR), naringenin 3-dioxygenase (F3H), and chalcone synthase (CHS) in ko00941 were significantly up-regulated in GS2 roots.

**Table 3:** KEGG pathway of DEGs in roots of common wild soybean seedlings under LP.

KO	KO ID	ID	FPKM		FC	Enzyme
			CK	LP		
**Phenylpropanoid biosynthesis**	ko00940	Glyma.03G208200.Wm82.a2.v1	18.17	44.04	1.28	cinnamyl-alcohol dehydrogenase (CAD)
		Glyma.09G277900.Wm82.a2.v1	3.26	7.95	1.02
		Glyma.10G022500.Wm82.a2.v1	12.92	29.93	1.04	peroxidase
		Glyma.18G055400.Wm82.a2.v1	4.14	17.55	1.62
		Glyma.18G055500.Wm82.a2.v1	6.37	28.96	1.25
**Pentose phosphate pathway**	ko00030	Glyma.11G111400.Wm82.a2.v1	1.10	–	-2.65	6-phosphofructokinase (PFK)
		Glyma.02G202500.Wm82.a2.v1	4.66	1.49	-1.36	triosephosphate dehydrogenase (gapN)
**Glutathione metabolism**	ko00480	Glyma.11G024000.Wm82.a2.v1	5.16	1.82	-1.10	glutathione peroxidase (gpx)
**Glycerollipid metabolism**	ko00561	Glyma.03G078300.Wm82.a2.v1	1.52	3.73	1.10	sulfoquinovosyltransferase (SQD2)
**Pentose and glucuronate interconversions**	ko00040	Glyma.04G119100.Wm82.a2.v1	1.21	3.48	1.16	pectinesterase
		Glyma.06G314100.Wm82.a2.v1	0.34	1.40	1.16
		Glyma.06G319100.Wm82.a2.v1	0.95	2.99	1.31
		Glyma.19G212400.Wm82.a2.v1	0.66	2.93	1.39

**Table 4:** KEGG pathway of DEGs in roots of barren-tolerant wild soybean seedlings under LP.

KEGG_map	ko_ID	ID	FPKM		FC	Enzyme
			CK	LP		
**Phenylpropanoid biosynthesis**	ko00940	Glyma.08G145900.Wm82.a2.v1	4.42	11.24	1.30	cinnamyl-alcohol dehydrogenase (CAD)
		Glyma.14G221200.Wm82.a2.v1	105.85	247.13	1.11
		Glyma.02G008900.Wm82.a2.v1	0.58	2.59	1.75	peroxidase
		Glyma.02G052700.Wm82.a2.v1	15.09	36.00	1.24
		Glyma.02G151700.Wm82.a2.v1	2.36	10.08	1.92
		Glyma.02G233900.Wm82.a2.v1	3.57	15.98	1.95
		Glyma.02G234000.Wm82.a2.v1	4.05	19.59	2.13
		Glyma.02G234200.Wm82.a2.v1	8.94	55.05	2.47
		Glyma.03G038100.Wm82.a2.v1	0.49	1.66	1.30
		Glyma.03G039800.Wm82.a2.v1	2.36	11.75	1.92
		Glyma.03G208200.Wm82.a2.v1	8.58	39.84	2.14
		Glyma.05G103600.Wm82.a2.v1	111.04	302.51	1.44
		Glyma.06G302600.Wm82.a2.v1	41.15	228.92	2.44
		Glyma.06G302700.Wm82.a2.v1	2.88	17.52	1.81
		Glyma.08G162700.Wm82.a2.v1	0.64	3.27	1.59
		Glyma.09G022500.Wm82.a2.v1	64.71	194.37	1.43
		Glyma.09G022800.Wm82.a2.v1	75.04	201.51	1.33
		Glyma.09G023000.Wm82.a2.v1	144.66	313.17	1.05
		Glyma.09G057100.Wm82.a2.v1	2.31	5.18	1.09
		Glyma.09G284400.Wm82.a2.v1	1.61	4.62	1.32
		Glyma.09G284700.Wm82.a2.v1	15.75	35.92	1.10
		Glyma.10G022500.Wm82.a2.v1	5.44	21.92	1.90
		Glyma.10G222400.Wm82.a2.v1	30.65	143.38	2.08
		Glyma.11G161600.Wm82.a2.v1	1.67	8.56	2.00
		Glyma.12G101200.Wm82.a2.v1	27.27	96.36	1.79
		Glyma.13G172300.Wm82.a2.v1	0.86	1.84	1.01
		Glyma.13G346100.Wm82.a2.v1	7.30	26.51	1.52
		Glyma.14G201700.Wm82.a2.v1	57.16	162.20	1.46
		Glyma.14G221400.Wm82.a2.v1	2.93	10.01	1.58
		Glyma.15G028200.Wm82.a2.v1	2.28	10.72	1.94
		Glyma.20G114200.Wm82.a2.v1	6.87	17.09	1.09	cinnamate hydroxylase (CYP73A)
**Flavonoid biosynthesis**	ko00941	Glyma.02G158700.Wm82.a2.v1	75.53	199.02	1.37	flavanone 4-reductase (DFR)
		Glyma.02G048400.Wm82.a2.v1	2.14	17.74	2.58	flavanone synthase I (F3H)
		Glyma.02G048600.Wm82.a2.v1	1.39	13.81	1.60
		Glyma.01G091400.Wm82.a2.v1	1.45	6.66	1.74	chalcone synthetase (CHS)
		Glyma.01G228700.Wm82.a2.v1	505.56	1132.52	1.13
		Glyma.02G130400.Wm82.a2.v1	5.49	21.24	1.61
		Glyma.05G153200.Wm82.a2.v1	5.21	11.98	1.12
		Glyma.08G109200.Wm82.a2.v1	6.75	16.75	1.20
		Glyma.08G109400.Wm82.a2.v1	14.54	40.21	1.19
		Glyma.08G110500.Wm82.a2.v1	25.83	63.77	1.06
		Glyma.08G110700.Wm82.a2.v1	17.41	60.64	1.48
		Glyma.08G110900.Wm82.a2.v1	12.40	37.26	1.49
		Glyma.11G011500.Wm82.a2.v1	283.06	640.68	1.10
**Phenylalanine, tyrosine and tryptophan biosynthesis**	ko00400	Glyma.01G131100.Wm82.a2.v1	1.41	4.82	1.36	aspartate-prephenate aminotransferase (PAT)
		Glyma.11G238300.Wm82.a2.v1	0.84	3.97	2.00
		Glyma.06G235500.Wm82.a2.v1	0.58	2.27	1.62	tyrosine transaminase (TAT)
		Glyma.19G239800.Wm82.a2.v1	0.67	2.46	1.54	shikimate dehydrogenase (DHQ-SDH)
**Pentose phosphate pathway**	ko00030	Glyma.05G056400.Wm82.a2.v1	8.75	21.46	1.28	transaldolase (talA)
		Glyma.08G199800.Wm82.a2.v1	3.02	1.14	-1.20	6-phosphofructokinase (PFK)
		Glyma.10G194300.Wm82.a2.v1	7.69	2.79	-1.68
		Glyma.10G293500.Wm82.a2.v1	0.25	0.85	1.49	transketolase (tktA)
		Glyma.02G202500.Wm82.a2.v1	6.09	1.71	-1.73	triosephosphate dehydrogenase (gapN)
		Glyma.08G182300.Wm82.a2.v1	4.52	12.86	1.41	fructose-bisphosphatase (FBP)
**Glycine, serine and threonine metabolism**	ko00260	Glyma.04G104500.Wm82.a2.v1	5.10	11.39	1.18	phosphoglyceromutase (PGAM)
		Glyma.06G105600.Wm82.a2.v1	0.91	3.03	1.30
		Glyma.04G254300.Wm82.a2.v1	2.60	1.19	-1.01	glycine hydroxymethyltransferase (glyA)
**Glutathione metabolism**	ko00480	Glyma.04G020200.Wm82.a2.v1	20.13	83.51	1.71	ornithine decarboxylase (ODC1)
		Glyma.06G020300.Wm82.a2.v1	1.01	14.05	1.71
		Glyma.14G177200.Wm82.a2.v1	0.48	2.06	1.21	L-ascorbate peroxidase
		Glyma.02G149100.Wm82.a2.v1	1.24	3.66	1.10	glutathione peroxidase (gpx)
**Glycerollipid metabolism**	ko00561	Glyma.03G078300.Wm82.a2.v1	1.61	6.08	1.71	sulfoquinovosyltransferase (SQD2)
		Glyma.15G034100.Wm82.a2.v1	0.32	1.37	1.41	lysophosphatidic acid-acyltransferase (AGPAT3_4)
**Pentose and glucuronate interconversions**	ko00040	Glyma.03G215900.Wm82.a2.v1	0.34	3.30	1.94	pectinesterase
		Glyma.03G216000.Wm82.a2.v1	9.99	39.58	1.75
		Glyma.04G119100.Wm82.a2.v1	0.22	1.29	1.98
		Glyma.05G236800.Wm82.a2.v1	0.21	0.56	1.27
		Glyma.06G314100.Wm82.a2.v1	0.29	1.11	1.31
		Glyma.06G319100.Wm82.a2.v1	0.12	0.81	2.18
		Glyma.09G232100.Wm82.a2.v1	2.15	4.88	1.14
		Glyma.12G004400.Wm82.a2.v1	1.03	3.61	1.44
		Glyma.19G212500.Wm82.a2.v1	0.43	1.52	1.41
		Glyma.19G231400.Wm82.a2.v1	0.13	1.75	2.58
**Glycerophospholipid metabolism**	ko00564	Glyma.03G092800.Wm82.a2.v1	3.88	13.60	1.72	phospholipase C (PLC)

**Figure 3:**
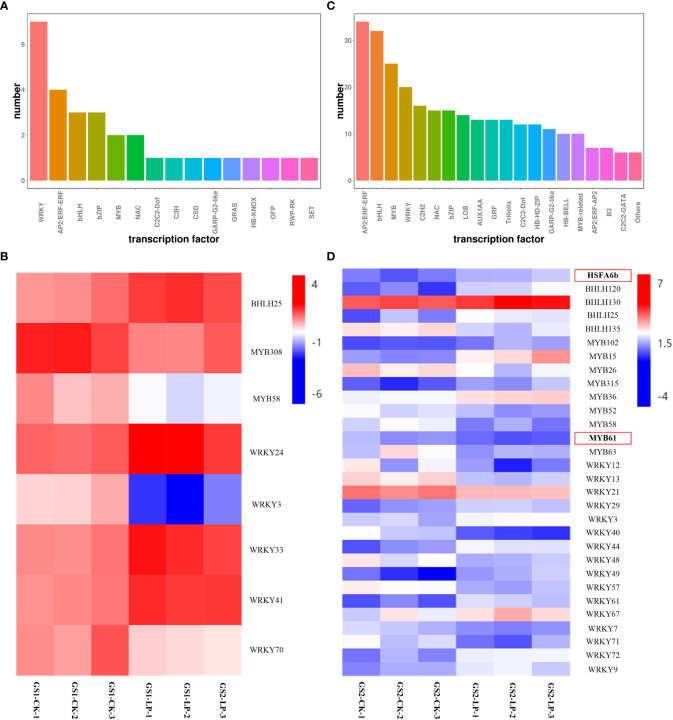
Differentially expressed transcription factor in roots of two ecotypes of wild soybean seedlings under control and low-phosphorus conditions. GS1, common wild soybean; GS2, barren-tolerant wild soybean; CK, control group; LP, low-phosphorus stress group. **(A)** Number of differentially expressed transcription factor in common wild soybean; **(B)** Regulation of the transcription factors in common wild soybean; **(C)** Number of differentially expressed transcription factor in barren-tolerant wild soybean; **(D)** Regulation of the transcription factors in barren-tolerant wild soybean. The expression levels represented as fold change; red indicates up-regulation; blue indicates down-regulation.

Transcription factor (TF) enrichment analysis showed that 30 DEGs were enriched in 15 TF families in GS1 roots, and 345 DEGs were enriched in 20 TF families in GS2 roots under LP stress (**Figure 3**; **Supplementary Material Table S5**). There were 1, 2, and 5 DEGs in the BHLH, MYB, and WRKY families in GS1 roots, respectively, and correspondingly 4, 9, and 16 DEGs in GS2 roots (**Figure 3C**). The *bHLH25*, *WRKY24*, *WRKY33* and *WRKY41* genes were specifically up-regulated 1.12-, 1.27-, 1.46- and 1.28-fold respectively; but *MYB308*, *MYB58*, *WRKY3*, *WRKY70* down-regulated 1.1-, 1.21-, 2.26- and 1.43-fold respectively in GS1 seedling roots. Genes *HSFA6b*, *BHLH120*, *BHLH130*, *BHLH25*, *MYB102*, *MYB15*, *MYB315*, *MYB36*, *WRKY29*, *WRKY3*, *WRKY44*, *WRKY49*, *WRKY61*, *WRKY67*, *WRKY72* and *WRKY9* were specifically up-regulated 1.29-, 1.82-, 1.17-, 1.39-, 1.21-, 2.8-, 1.4-, 1.05-, 1.09-, 1.07-, 1.7-, 1.5-, 1.75-, 1.03-, 1.7- and 1.12-fold respectively; but *BHLH35*, *MYB26*, *MYB52*, *MYB58*, *MYB61*, *MYB63*, *WRKY12*, *WRKY13*, *WRKY21*, *WRKY40*, *WRKY48*, *WRKY57*, *WRKY7* and *WRKY71* down-regulated 1.26-, 1.05-, 1.08-, 1.43-, 1.07-, 1.37-, 1.46-, 1.77-, 1.2-, 2.4-, 1.16-, 1.53-, 1.04- and 1.29-fold respectively in GS2 seedling roots. The expression of *HSFA6b* family gene Glyma.10G244000.Wm82.a2.v1 and the *MYB61* family gene Glyma.19G222200.Wm82.a2.v1 were significantly changed in GS2 seedling roots, but not detected in GS1 roots (**Figure 3D**; **Supplementary Material Table S5**).

### 3.3 Changes in metabolic levels

**Figure 4:**
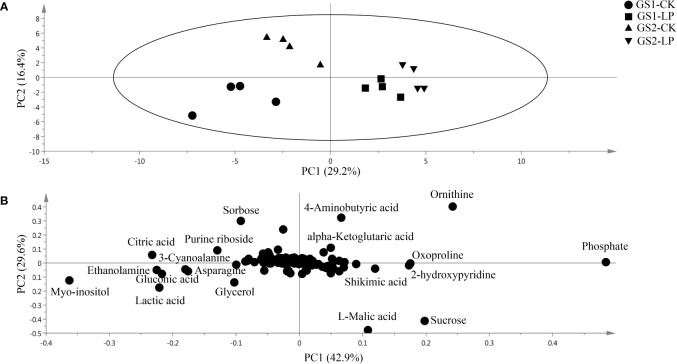
**(A)** Principal component analysis and **(B)** loading plots of metabolites in roots of two ecotypes of wild soybean seedlings under control and low-phosphorus conditions. PC1 and PC2 are the first and second principal components, respectively. GS1, common wild soybean; GS2, barren-tolerant wild soybean; CK, control group; LP, low-phosphorus stress group.

Based on GC-MS, we conducted metabonomics studies on the seedling roots of two wild soybean ecotypes under LP stress (**Supplementary Material Figure S5**). Metabolomics PCA showed that the first principal component (PC1) explained 29.2% of the variation (**Figure 4A**), showing significant differences in the metabolomics for GS1 and GS2 roots in CK and LP stress. The second principal component (PC2) explained 16.4% of the variation (**Figure 4A**), among which the difference in metabonomics between CK and LP stress of GS1 roots was greater than that in GS2. The PLS-DA showed that the main contributors to PC1 were myo-inositol, ethanolamine, gluconic acid, citric acid, 3-cyanoalanine, lactic acid, ornithine, oxoproline, shikimic acid and phosphate; and the main contributors to PC2 were ornithine, L-malic acid, sucrose and 4-aminobutyric acid (**Figure 4B**; **Supplementary material Table S6**). There were 50 different metabolites screened according to *p* < 0.05, similarity ≥ 600, and VIP > 1 from the measured metabolites: 12 amino acid metabolites, 12 sugar alcohol metabolites, 18 organic acid metabolites, and eight secondary metabolites.

**Figure 5:**
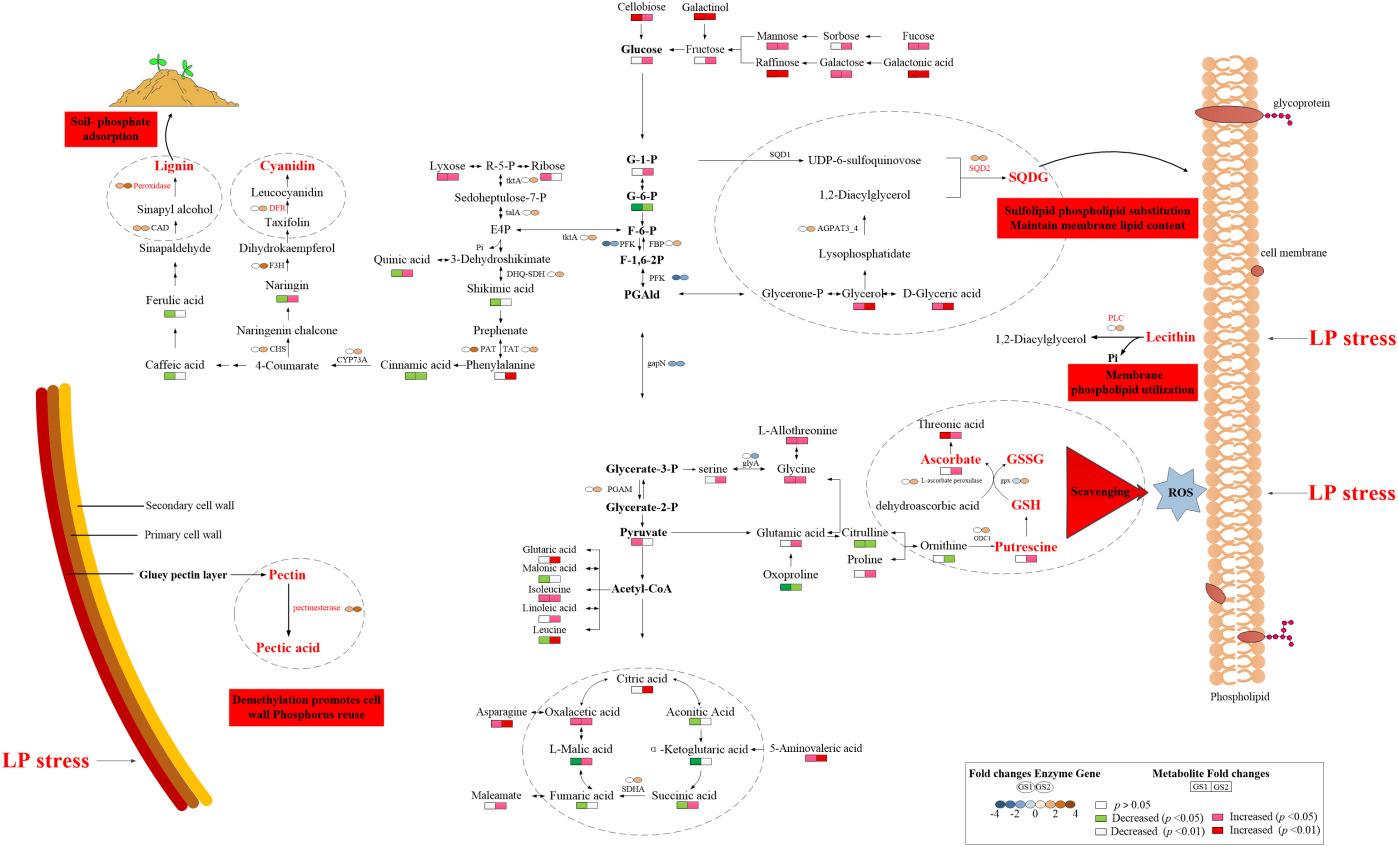
Key mechanisms of resistance to phosphorus deficiency in wild soybean. GS1, common wild soybean; GS2, barren-tolerant wild soybean; AGPAT3_4, lysophosphatidic acid acyltransferase; CAD, cinnamyl-alcohol dehydrogenase; CHS, chalcone synthase; CYP73A, trans-cinnamate 4-monooxygenase; DFR, bifunctional dihydroflavonol 4-reductase/flavanone 4-reductase; DHQ-SDH, 3-dehydroquinate dehydratase/shikimate dehydrogenase; F3H, naringenin 3-dioxygenase; FBP, fructose-1,6-bisphosphatase; gapN, glyceraldehyde-3-phosphate dehydrogenase (NADP^+^); glyA, glycine hydroxymethyltransferase; gpx, glutathione peroxidase; GSH, glutathione; GSSG, glutathione disulfide; ODC1, ornithine decarboxylase; PAT, aspartate-prephenate aminotransferase; PFK, 6-phosphofructokinase; PGAM, 2,3-bisphosphoglycerate-dependent phosphoglycerate mutase; PLC, phospholipase C; SDHA, succinic dehydrogenase, SQD2, sulfoquinovosyltransferase; talA, transaldolase; TAT, tyrosine aminotransferase; tktA, transketolase.

The responses of metabolites in the two ecotypes to LP stress significantly differed (**Table 5**; **Figure 5**). Lignin and the anthocyanin synthesis key metabolite, phenylalanine, increased 1.76-fold in GS2 (*p* < 0.01), but with no significant difference in GS1 roots (*p* > 0.05). Naringin related to anthocyanin synthesis decreased 0.17-fold in GS1 roots, but increased 0.66-fold in GS2 (*p* < 0.05). Related to the GSH-GSSG and AsA–GSH cycles, with functions of scavenging ROS metabolites, proline, serine, glutamic acid, putrescine, and ascorbate increased 0.08-, 0.24-, 0.75-, 1.88-, and 1.06-fold, respectively, in GS2 roots (*p* < 0.05), but there was no significant change in GS1 (*p* > 0.05). Under LP stress, sugar alcohol metabolites accumulated in roots of both ecotypes. Raffinose and cellobiose significantly increased by 1.39- and 0.74-fold (both *p* < 0.05) in GS1 roots, respectively, and significantly by 1.80- and 0.88-fold (both *p* < 0.01) in GS2. The sugar alcohol metabolism under LP stress was more enhanced in GS2 roots than GS1 compared with CK. Under LP stress, the response of organic acid metabolites in GS1 and GS2 roots was very different. Citric acid significantly increased by 0.70-fold (*p* < 0.01) and succinic, L-malic, and oxalacetic acids increased by 0.07-, 0.46-, and 1.63-fold (all *p* < 0.05), in GS2 roots under LP stress. Aconitic, succinic, fumaric, and L-malic acids significantly decreased by 3.74-, 0.27-, 0.48-, and 0.44-fold in GS1 roots, respectively (all *p* < 0.05), and α-ketoglutaric acid significantly decreased by 2.42-fold (*p* < 0.01). This showed that the TCA cycle was enhanced in GS2 roots and inhibited in those of GS1. Glycerol, involved in sulfoquinovosyl diacylglycerol (SQDG) synthesis significantly increased by 0.45-fold (*p* < 0.05) and 0.57-fold (*p* < 0.01) in GS1 and GS2 roots, respectively.

**Table 5:** Changes of metabolite contents of two ecotypes wild soybean seedling roots under LP.

Metabolite name		Relative concentration				FD	
		GS1		GS2			
		CK	LP	CK	LP	GS1	GS2
	Oxoproline	18.82	8.78	15.98	10.34	-1.10**	-0.63*
	Proline	1.33	1.52	1.6	1.69	0.19	0.08*
	Glycine	1.3	2.67	1.89	2.12	1.04*	0.17*
	Serine	4.71	6.36	6.49	7.65	0.43	0.24*
	Glutamic acid	0.15	0.14	0.14	0.23	-0.13	0.75*
Amino acid metabolites	Ornithine	0.49	0.36	0.74	0.5	-0.44	-0.57*
	Phenylalanine	0.35	0.88	0.31	1.04	1.32	1.76**
	Citrulline	2.15	1.4	1.71	1.28	-0.62*	-0.42*
	Asparagine	1.14	12.55	1.21	9.08	3.46*	2.91*
	Leucine	0.38	0.22	0.84	4.22	-0.81*	2.33*
	Isoleucine	1.77	2.9	2.3	3.42	0.71*	0.57*
	L-Allothreonine	8.27	10.21	8.65	11.15	0.30*	0.37*
Sugar alcohol metabolites	Sorbose	37.35	36.65	51.33	68.03	-0.03	0.41*
	Glucose	1.79	2.45	3.53	4.19	0.45	0.25*
	Fructose	25.16	21.39	34.02	41.12	-0.23	0.27*
	Ribose	2.68	5.75	4.43	5.33	1.10*	0.27
	Raffinose	0.86	2.25	0.99	3.46	1.39*	1.80**
Organic acid metabolites	Lyxose	0.62	0.79	0.76	1.19	0.35*	0.66*
	Cellobiose	3.36	5.63	4.22	7.75	0.74*	0.88**
	Galactose	0.09	0.24	0.16	0.28	1.42*	0.78*
	Mannose	0.09	0.17	0.15	0.2	0.99*	0.45*
	Galactinol	1.12	3.3	0.83	3.91	1.56**	2.24**
	Fucose	0.14	0.17	0.19	0.24	0.30*	0.34*
	Glycerol	11.39	15.52	8.09	12.01	0.45*	0.57**
	Glucose-6-phosphate	1.5	0.13	1.31	0.21	-3.56**	-2.62*
	Glucose-1-phosphate	5.7	6.06	5.93	6.26	0.09	0.08*
	Pyruvate	0.53	0.84	0.73	0.81	0.65*	0.16
	Citric acid	44.64	57.14	44.6	72.38	0.36	0.70**
	Aconitic acid	0.34	0.03	0.01	0.03	-3.74*	-2.02
	α-Ketoglutaric acid	0.14	0.03	0.09	0.04	-2.42**	-1.28
	Succinic acid	16.38	13.59	13.19	13.85	-0.27*	0.07*
	Fumaric acid	5.05	3.63	3.97	3.46	-0.48*	-0.2
	L-Malic acid	126.78	93.23	59.91	82.13	-0.44**	0.46*
	Oxalacetic acid	0.18	0.36	0.14	0.44	1.01*	1.63*
	5-Aminovaleric acid	2.85	4.36	2.98	5.67	0.61*	0.93**
	Maleamate	0.02	0.03	0.01	0.11	0.61	4.04*
	Galactonic acid	0.29	0.69	0.52	1.04	1.26**	1.00**
	Threonic acid	0.7	1.37	0.75	2.29	0.96**	1.62*
	Malonic acid	3.35	2.26	2.44	2.53	-0.57*	0.05
	Glutaric acid	0.87	0.56	0	0.08	-0.63	11.63**
	Linoleic acid	0.27	0.47	0.23	0.59	0.79	1.39*
	D-Glyceric acid	0.15	0.36	0.19	0.3	1.28*	0.69**
	Putrescine	0.62	0.76	0.36	1.32	0.29	1.88*
	Ascorbate	0.04	0.06	0.03	0.07	0.52	1.06*
	Shikimic acid	0.48	0.45	0.63	0.55	-0.10*	-0.2
	Quinic acid	0.04	0.03	0.03	0.03	-0.83*	0.23*
Secondary metabolites	Cinnamic acid	1.82	1.03	2.11	1.22	-0.82*	-0.80*
	Naringin	0.03	0.02	0.27	0.42	-0.17*	0.66*
	Ferulic acid	0.17	0.1	0.18	0.11	-0.73*	-0.69
	Caffeic acid	0.04	0.02	0.05	0.02	-1.30*	-1.12

Relative concentration values were presented as the mean of four biological replicates. Relative concentration values were increased 1000 times. The * and ** indicate significant (P<0.05) and highly significant difference (P<0.01), respectively. FD, fold changes that the change of growth parameters is represented by log_2_^(LP/CK)^.

### 3.4 Integrated analysis of transcriptome and metabolomics in barren-tolerant wild soybean

**Figure 6:**
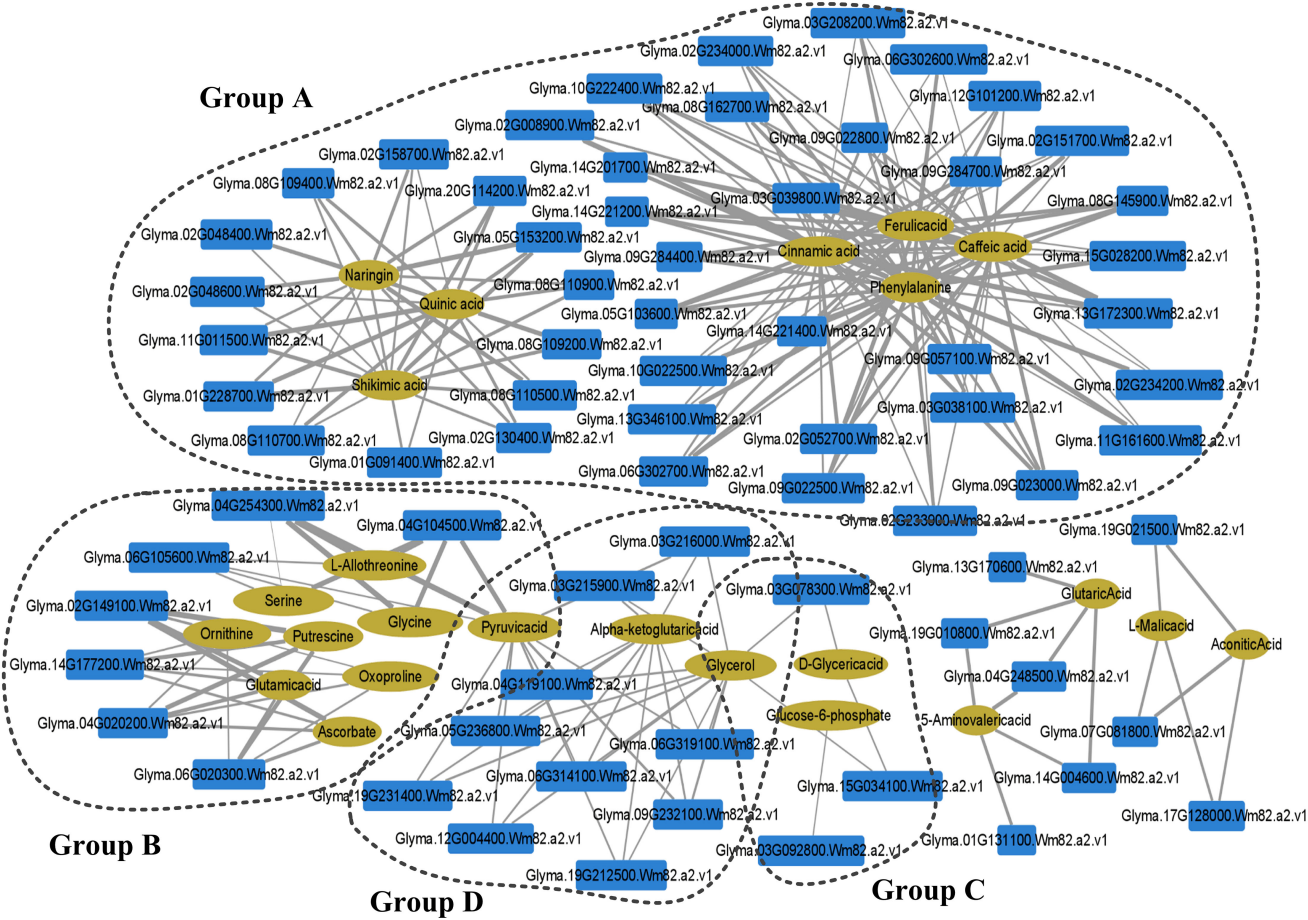
Integration network of metabolites and genes in barren-tolerant wild soybean seedling roots under phosphorus deficiency. The thicker the edge, the stronger the correlation. The size of a node is proportional to the correlation between nodes. Group A, closely related to secondary metabolism; Group B, related to remove ROS and protect the stability of cell membranes; Group C, related to membrane lipid remodeling and membrane phospholipid breakdown.

In order to determine the key regulatory network mechanism of GS2 tolerance to LP stress, we mapped 50 different metabolites and 2948 DEGs to the KEGG database, and performed Pearson correlation analysis in GS2 seedling roots under LP stress (**Supplementary Material Table S7**). The correlation interaction networks in GS2 roots showed that 76 transcripts had high correlation coefficients with 22 metabolites (**Figure 6**; **Supplementary Material Table S7**). The networks showed that the transcripts and metabolites were in four groups (A-D).

Group A was closely related to secondary metabolism and included phenylpropanoid biosynthesis (Ko00940); flavonoid biosynthesis (Ko00941); phenylalanine, tyrosine, and tryptophan biosynthesis (Ko00040); and pentose phosphate pathway (Ko00030) related transcripts and metabolites. Among them, phenylalanine and ferulic, caffeic, and cinnamic acids were significantly related to the DEGs of CAD, peroxidase, and cinnamate hydroxylase (CYP73A), which are involved in lignin synthesis. The flavonoid metabolites naringin, quinic acid, and shikimic acid were significantly related to the DEGs of DFR, F3H, and CHS, which are the key enzymes for anthocyanin synthesis of secondary antioxidants. Group B included glycine, serine, and threonine metabolism (Ko00260) and glutathione metabolism (Ko00480) which can remove ROS and protect the stability of cell membranes. Putrescine and ascorbic acid were significantly related to the DEGs of L-ascorbate peroxidase and gpx in the AsA–GSH cycle. Group C was related to membrane lipid remodeling and membrane phospholipid breakdown, including glycerolipid metabolism (Ko00561) and glycerophospholipid metabolism (Ko00564). The metabolites glycerol and D-glyceric acid were significantly related to the gene encoding SQD2, a key enzyme in SQDG synthesis. The Pearson’s correlation coefficient between glucose-6-phosphate and the gene encoding PLC was −0.85306. Group D was related to the hydrolysis of pectin in the pectin layer of the cell wall that includes pentose and glucuronate interconversions (Ko00040), in which glycerol, pyruvate, and α-ketoglutarate were significantly negatively correlated with genes encoding pectinesterase, the key enzyme for pectin hydrolysis, with Pearson’s correlation coefficients between −0.35914 and −0.8638 (Ko00040) (**Figure 6**).

## 4 Discussion

The adaptation of plants to their environment is due to the variation of certain physiological and biochemical functions in the plant. These variations are due to mutations in the genetic material that cause differences within the one species, forming different ecotypes adapt to different environments (Ellis et al., 2021; Postolache et al., 2021). Therefore, the causes of variation from multiple perspectives, such as morphological structure, physiology, metabolism, and molecules, can better reveal the mechanisms of ecological adaptation formation during plant evolution.

The smaller inhibition of growth and the smaller decrease of P content in GS2 roots with LP compared to GS1 suggests that GS2 is more tolerant to soil P deficiency and has more free-P. In general, LP-resistant ecotypes of plants can enhance external P uptake and adjust internal P reuse to better adapt to P deficient environments by modulating morphological structures, as well as genetic and metabolic changes. Studies have confirmed that plants can expand the contact area with soil P through increasing the number and length of root hairs and lateral roots, increasing the root-to-shoot ratio, and the production of cluster roots, thereby increasing the plant’s absorption of P (Watanabe et al., 2006; Niu et al., 2012; Yuan et al., 2016). In this study, the growth of GS1 plants was inhibited by P deficiency, and both plant height and root length decreased. The plant height of GS2 was reduced, but root length significantly increased, showing that in order to resist LP stress, GS2 plants distributed a larger proportion of material and energy to root growth, which greatly increased the root–soil contact area and so enhanced P absorption. Enhanced expression of the genes encoding PHT1 could promote P transport in plants, so as to maintain P homeostasis, and better respond to P deficiency (Fan et al., 2013; Song et al., 2014). Zhang et al., 2014 The purple acid phosphatase in plants is induced by P deficiency and could hydrolyze and release P from organophosphorus substrates for plant uptake and reuse. Low-P tolerant plants could significantly induce the purple acid phosphatase gene expression under P deficiency, so as to synthesize more purple phosphatase to promote P uptake and reuse, thus maintaining stable P content and better responding to low-P stress (Li et al., 2012). At the same time, the lignin in plant roots can significantly reduce the soil’s ability to adsorb and fix P, and increase availability of P in the soil (Zhang, 2006). The key enzyme of lignin synthesis, peroxidase, and CAD-related DEGs were up-regulated, phenylalanine significantly accumulated, phenol metabolism was enhanced, and more P could be obtained in GS2 under LP stress. Transcriptomics GO annotation of DEGs also confirmed the significant enrichment of the flavonoid biosynthetic process (GO:0009813) and positive regulation of the flavonoid biosynthetic process (GO:0009963). Most of the insoluble inorganic P in the soil is bound by Al^3+^, Fe^3+^, and Ca^2+^ to form insoluble compounds; however, the P can be replaced by organic acid anions secreted by roots, releasing more available P for plants to use (Shane and Lambers, 2006). Significant accumulation of organic acids and rhizosphere secretion of organic acids have been detected in the roots of different species grown under low-P stress positive correlations were found (Haichar et al., 2014). Thus plants can resist low-P stress by increasing the content of organic acids in roots and then secreting more organic acids to the outside to increase the content of available P in plants (Lipton et al., 1987). The contents of citric and L-malic acids increased significantly, indicating that GS2 could effectively regulate the accumulation of organic acids in roots under low-P stress, which is beneficial to its secretion of organic acids to activate unavailable P in soil to resist low-P stress. Transcriptomic GO annotation of DEGs also confirmed the significant enrichment in carboxylic acid metabolic process (GO:0019752). *Lupinus albus* and *Vicia faba* can secrete large amounts of organic acids to enhance the efficiency of P acquisition under LP stress (Shen et al., 2018). Organic acid metabolism was significantly enhanced in GS2, showing that this was an important mechanism of GS2 to deal with LP stress. However, the metabolism of organic acids in GS1 changed irregularly under the influence of LP stress.

In addition to enhancing absorption of P, improving the utilization efficiency of P and structural P reuse in plant cells, such as hydrolyzing non-organic P in senescent tissues and being used by new tissues, is an important mechanism for plants to tolerate P deficiency in soil (Gong et al., 2011). Pectinesterase can maintain the degree of pectin methyl esterification of the cell wall at a low level, thereby promoting the release of cell wall P to increase the soluble P content in the body for reuse in other parts (Micheli, 2001; Zhu et al., 2012). Under LP stress, DEGs were significantly enriched in the pectin catabolic process (GO:0045490), and the pectinesterase-related DEGs in the two ecotypes of wild soybean were up-regulated, and the greater changes in GS2 indicated that GS2 could better actively regulate the reuse of endogenous P in the cell wall and resist soil P deficiency. Membrane phospholipids absorb one-third of the P in plant cells, when phosphate is insufficient, and PLC can hydrolyze phospholipids and promote the mobilization of organic phosphate in membrane phospholipids for other physiological metabolic activities (Tjellström et al., 2010). At the same time, PLC is also an important regulatory enzyme in the process of abiotic stress response (Peters et al., 2014). The PLC-related DEGs were up-regulated, showing that active regulation of membrane lipid metabolism is one key mechanism for GS2 to respond to LP stress, while this phenomenon does not exist in GS1. Accompanied by changes in membrane lipid metabolism, it is necessary to improve the structural stability of membrane lipids in cells to resist LP stress. Under P-limited conditions, the content of phosphatidylglycerol (PG) in *Arabidopsis* membrane lipids decreased, and the content of sulfur-containing glycolipid SQDG increased, replacing PG with SQDG to maintain stable membrane lipid content and membrane lipid structure. Consistent with previous studies, the accumulations of D-glyceric acid and glycerol were significantly related to the up-regulated gene for SQDG, showing that SQDG activity was enhanced, maintaining the structural integrity of the biofilm system under LP stress in GS2. This revealed that the key mechanism to resisting LP stress was the decomposition and reuse of structural P, at the same time maintaining the stable structure and function of the cell membrane in GS2. In addition, enhanced sugar metabolism in plants facilitates the reduction of phosphorylation of metabolites to P-free sugars for efficient P use. Metabolomics research found that, under LP stress, the content of UDP-glucose in *Arabidopsis* leaves and di- and tri-saccharides in maize shoots accumulated and so enhanced the ability of plants to resist soil P deficiency (Ciereszko and Kleczkowski, 2006; Ganie et al., 2015). In this study, the disaccharides raffinose and cellobiose showed extremely significant accumulation, the sugar alcohol metabolism of GS2 was enhanced and its ability to withstand soil P deficiency was improved, consistent with previous studies. Sugar alcohol metabolism was enhanced in GS1, but the increase was not as great as GS2. In addition, citric and L-malic acids are important metabolites in the TCA cycle, and oxalacetic and succinic acids also accumulated significantly, showing that the TCA cycle was enhanced to provide energy for plant metabolic activities in GS2 under LP stress.

Under P deficiency, plants will produce ROS to initiate membrane lipid peroxidation, leading to membrane damage and destruction. One plant phenotype in response to soil P deficiency is the accumulation of anthocyanins in tissues, which can scavenge free radicals under adverse conditions and increase plant tolerance to P deficiency (Liang and He, 2018; He et al., 2020). Transcriptomics and metabolomics correlation analysis showed that genes related to DFR, a key enzyme for anthocyanin synthesis, were significantly up-regulated, and naringin accumulated significantly in GS2. The GSH is an important antioxidant and free radical scavenger. Under the catalysis of gpx, toxic peroxides are reduced to non-toxic compounds, and at the same time, GSH becomes GSSG. In the AsA–GSH cycle, AsA significantly accumulated, gpx-related DEGs were significantly up-regulated, and enhancement of the AsA–GSH cycle could effectively remove free radicals produced by LP stress and inhibit stress-induced oxidative damage in GS2. The AsA–GSH cycle also had a similar response when *Cunninghamia lanceolata* and *Oryza sativa* were subjected to soil P deficiency (Luo et al., 2018).

In the signal transduction system of plants, TFs play an important role in response to adverse environments. It has been reported that TFs of the BHLH, MYB, and WRKY families are closely related to the regulation of gene expression under P deficiency (Liang et al., 2013; Fan et al., 2014). In this study, two ecotypes of wild soybean were enriched in the WRKY, BHLH, and MYB families under LP stress, especially GS2. It is noteworthy that the HSFA6b gene was significantly up-regulated, and the MYB61 gene was significantly down-regulated in GS2 seedling roots. Previous studies have shown that HSFA6b and MYB61 play an important role in LP stress in Arabidopsis thaliana (Liang et al., 2005; Romano et al., 2012; Huang et al., 2016). Previous studies have shown that the MYB61 gene is essential for lignin biosynthesis (Rahmawati et al., 2022). The key enzyme of lignin synthesis, peroxidase, and CAD-related DEGs were up-regulated, further demonstrating that the ability of MYB61 to improve the ability of GS2 to resist low phosphorus stress may be related to the promotion of lignin synthesis.

## 5 Conclusions

Due to variation in the environment, the adaptation of plants to the environment is in a constant process of regulation. This coordination between different ecotypes of plants and environments can be achieved by plants changing their morphological structure, physiology, and molecular metabolism. In this study, through morphological and integrated analysis of transcriptomics and metabolomics of the roots of two ecotypes of wild soybean seedlings, on the one hand, GS2 enhanced soil P uptake by increasing root length, promoting the synthesis and secretion of lignin and organic acids and upregulating inorganic phosphate transporter (PHT1-2 and PHT1-3), acid phosphatase ACP1, and purple acid phosphatase genes (PAP36, PAP14 and LOC100782755) promote P uptake and transport to maintain stable P content in plants, and thus better adapt to low-P stress. On the other hand, GS2 increased pectin esterase and PLC activities to promote the reuse of structural P in the cell wall and membrane lipids, respectively, to maintain the P source required for normal cellular activities. In addition, GS2 generated SQDG to replace PG and maintain the stability of biofilm structure, and enhanced the synthesis of secondary antioxidant metabolite anthocyanins and the AsA–GSH cycle to reduce the oxidative damage to the membranes caused by P deficiency in soil. It was also found that HSFA6b and MYB61 were key TFs for barren-tolerant wild soybean to resist LP stress. Our study provides new perspectives for the divergence evolution of plants and provides quantitative system parameters for resource evaluation.

## Data availability statement

The original contributions presented in the study are publicly available. This data can be found here: NCBI, PRJNA865466.

## Author contributions

ML and LS designed the study and methodology. JZ, XL, DH, YH, YD and GW performed the analyses and discussed the results. ML wrote the manuscript draft. JG and LS performed writing-review, editing and supervision. All authors contributed to the article and approved the submitted version.

## Funding

This work was supported by the National Natural Science Foundation of China (No. 32072012), Natural Science Foundation of Jilin Province, China (No. 20200201134JC), and Natural Science Foundation of Jilin Province, China (No. 20220508057RC).

## Acknowledgments

We thank International Science Editing (http://www.internationalscienceediting.com) for editing this manuscript.

## Conflict of interest

The authors declare that the research was conducted in the absence of any commercial or financial relationships that could be construed as a potential conflict of interest.

## Publisher’s note

All claims expressed in this article are solely those of the authors and do not necessarily represent those of their affiliated organizations, or those of the publisher, the editors and the reviewers. Any product that may be evaluated in this article, or claim that may be made by its manufacturer, is not guaranteed or endorsed by the publisher.

## Abbreviations

GS2, barren-tolerant wild soybean; GS1, common wild soybean; PLC, phospholipase C; SQD2, the key enzyme of sulfolipid substitution of phospholipids; P, Phosphorus; LP, low- phosphorus; ROS, reactive oxygen species; CAD, cinnamyl-alcohol dehydrogenase; GSH-GSSH, glutathione-glutathione disulfide; gpx, glutathione peroxidase; AsA, ascorbate; DFR, flavanone 4-reductase; F3H, naringenin 3-dioxygenase; CHS, chalcone synthase; TF, Transcription factor; PC1, the first principal component; PC2, The second principal component; SQDG, sulfoquinovosyl diacylglycerol; CYP73A, cinnamate hydroxylase; CK, control; DEGs, differentially expressed genes; GC/MS, gas chromatograph-mass spectrometer; PCA, principal component analysis; OPLS-DA, orthogonal partial least-squares discrimination analysis; PLS-DA, partial least squares discriminant analysis.
