# Supplementary material for: Integrating transcriptomic and metabolomic analysis in roots of wild soybean seedlings in response to low-phosphorus stress
Source: Front Plant Sci. 2022 Nov 17;13:1006806. doi: 10.3389/fpls.2022.1006806 (PMC9713585; doi:10.3389/fpls.2022.1006806)
Supplement: Supplementary file 8 [file DataSheet_8.docx]

Supplementary Material





**Supplementary Figure 1.** qRT-PCR of 13 genes in roots of two ecotypes of wild soybean seedling.





**Supplementary Figure 2.** DEGs of wild and cultivated soybean seedling roots under low-phosphorus stress and control: (a) volcano and (b) Venn diagrams.





**Supplementary Figure 3.** GO annotation of DEGs in roots of two ecotypes of wild soybean seedlings.





**Supplementary Figure 4.** KEGG enrichment of DEGs in (a) common and (b) barren-tolerant wild soybean.





**Supplementary Figure 5.** Total ion current chromatograms of extracts of roots of two ecotypes of wild soybean seedlings obtained from GC-MS: (A) GS1-CK, (B) GS1-LP, (C) GS2-CK, and (D) GS2-LP.
